# Supplementary material for: A comparison of respiratory particle emission rates at rest and while speaking or exercising
Source: Commun Med (Lond). 2022 Apr 19;2:44. doi: 10.1038/s43856-022-00103-w (PMC9053213; doi:10.1038/s43856-022-00103-w)
Supplement: Supplementary file 1 — Supplementary Information [file 43856_2022_103_MOESM1_ESM.pdf]

A comparison of respiratory particle emission rates at rest and while speaking or exercising

Christopher M. Orton†,[1,2,3] Henry E. Symons†,[4] Benjamin Moseley,[1] Justice Archer,[4] Natalie A. Watson,[5] Keir E.J. Philip, [1,3] Sadiyah Sheikh, [4] Brian Saccente-Kennedy, [6] Declan Costello,[7] William J. Browne, [8] James D. Calder,[9,10] Bryan R. Bzdek, [4] James H. Hull,[1,11] Jonathan P. Reid*,[4] Pallav L. Shah* [1,2,3]

[1]Department of Respiratory Medicine, Royal Brompton Hospital, London, UK

[2]Department of Respiratory Medicine, Chelsea & Westminster Hospital, London, UK

[3]National Heart and Lung Institute, Guy Scadding Building, Imperial College London, Dovehouse Street, London, UK

[4]School of Chemistry, University of Bristol, Bristol, UK

[5]Department of Ear, Nose and Throat Surgery, Guys & St. Thomas NHS Foundation Trust, London, UK

[6]Speech and Language Therapy Department, Royal National Ear Nose and Throat Hospital, London, UK

[7]Ear, Nose and Throat Department, Wexham Park Hospital, UK

[8]School of Education, University of Bristol, Bristol, UK

[9]Department of Bioengineering, Imperial College London, UK

[10]Fortius Clinic, Fitzhardinge St, London, UK

[11]Institute of Sport, Exercise and Health (ISEH), UCL, London, UK

†These authors contributed equally.

*Corresponding authors: pallav.shah@imperial.ac.uk, j.p.reid@bristol.ac.uk and c.orton@rbht.nhs.uk

Supplementary Information

Supplementary methods 1 Aerosol Sampling Methodology

For participants E1-E10 we explored suitable methods to sample aerosol during exercise, specifically: the approach previously reported, where a 3D-printed cone connected by conductive tubing to an APS is placed in close proximity to the participant's nose and mouth (APS-cone, see Supplementary Figure 1)[1], and an alternative method where a sampling tube was inserted directly into the participant's CPET mask (APS-mask, see Figure 1a). We compare aerosol concentrations for participants speaking at a level of 70-80 dBA by each method to those reported during the same activity in a recent study on respiratory aerosol (see Supplementary Figure 2)[1]. Sampling via APS-mask gave median number and mass concentrations of 0.26 cm^{-3} (IQR 0.21-0.31) and 0.40 µg m^{-3} (IQR 0.25-1.22) across all 25 participants. When mean data for each of the 25 participants were compared to data from a previously reported cohort of 25 singers, neither number concentration (two-sample $t(48)=1.4$, $p=0.154$) nor mass concentration (two-sample $t(48)=1.9$, $p=0.060$) were different to those (median values of 0.22 cm^{-3} IQR 0.14-0.27 and 0.23 µg m^{-3} IQR 0.20-0.54, respectively) reported previously[1]. In contrast, the concentrations recorded for participants E1-E10 via APS-cone were lower than those reported for the cohort of 25 singers[1], two-sample $t(33)=10.5$, $p<0.001$ and two-sample $t(33)=5.9$, $p<0.001$ for number and mass concentrations, respectively, with median values of 0.019 cm^{-3} (IQR 0.011-0.021) and 0.026 µg m^{-3} (IQR 0.023-0.087). It is likely that the large distance between the participant and the cone is a key factor in the reduced particle concentration recorded. We have provided a comprehensive account of the challenges of making measurements of respiratory aerosol using the approaches described here, along with measurement uncertainties and limitations, in a recent publication[2].

Average size distributions obtained by both methods are compared to previous reported data (see Supplementary Figure 3). Almost all data points for the average size distribution for speaking at 70 – 80 dBA lie within the error bounds of those previously reported when recorded inside the mask[1,3]. When compared with measurements reported previously, the data are consistent through a constant scaling factor accounting for sampling efficiency differences. As further confirmation of the suitability of this sampling methodology, mass concentrations whilst speaking at 70 – 80 dBA were calculated using both the size distributions obtained here

and those reported by previously by the authors, alongside the number concentrations recorded in this study[1,3]. The concentrations obtained for all 25 participants using the previously reported size distributions (median value of 0.41 µg m^{-3} IQR 0.33-0.49) were no different (two-sample t(48)=1.2, p=0.25) to those reported in the main text for the same participants (median 0.40 µg m^{-3} IQR 0.25-1.22).

Based on these findings, sampling within the CPET mask allowed the greatest comparability with previous studies and minimised sampling errors arising from the challenges of participants continuously aligning the CPET mask to the sampling cone and maintaining a constant close distance (<10 cm) throughout the measurement sequence.

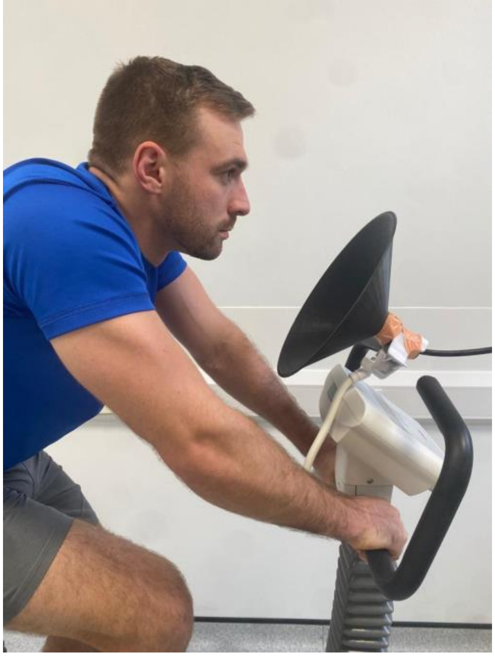

Supplementary Figure 1. ***APS-cone experimental setup.*** *Experimental setup for aerosol data recorded via APS-cone. Sampling cone was adjusted to a distance of approximately 15 cm from each participants mouth. Informed consent for publication of the image was obtained.*

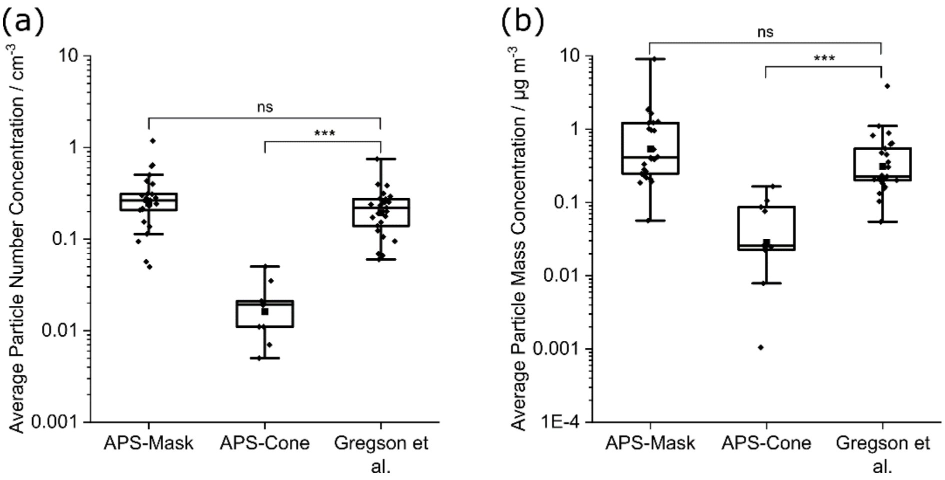

Supplementary Figure 2 ***Aerosol number and mass concentration validation during speaking.*** Box and whisker plots comparing number (a) and mass (b) concentrations of aerosol produced by speaking at 70-80 dBA, measured via APS-mask, or via APS-cone, with previously reported data[1], *across n=25 participants*. Boxes indicate mean, median and IQR, whiskers indicate range (data within 1.5 IQR), *** indicates p<0.001, not significant (ns).

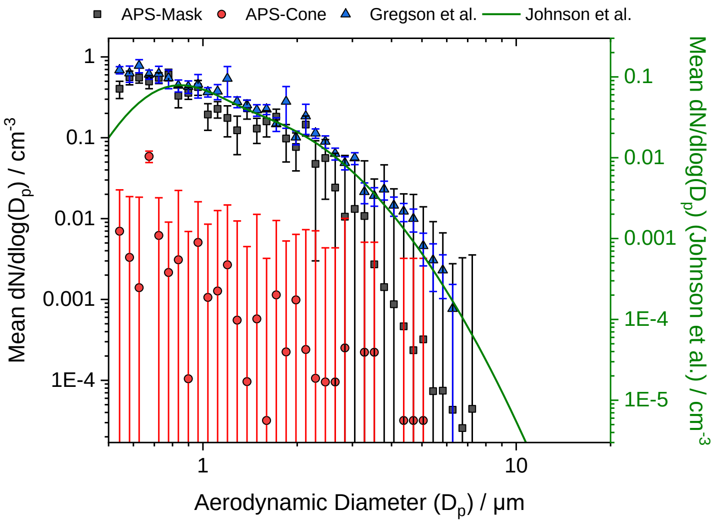

Supplementary Figure 3. ***Aerosol size distribution validation.*** *Comparison of average size distributions from speaking at 70-80 dBA measured via APS-mask (black squares), or via APS-cone (red circles) across n=25 participants, with previously reported data (blue triangles)* [1,3]*. Error bars represent the standard error of the mean.*

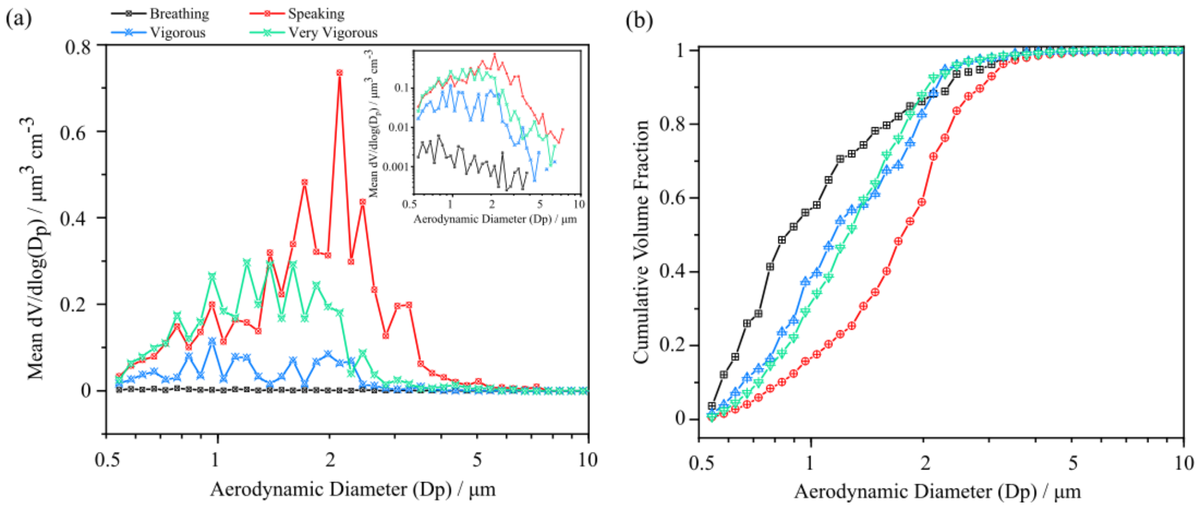

*Supplementary Figure 4 :**Volume Aerosol size distributions for breathing at rest, speaking, vigorous exercise and very vigorous exercise.** n=25 participants. (a)Comparison of average volume size distributions during breathing at rest (dark grey), speaking (red), vigorous exercise (blue) and very vigorous exercise (green). (a)Inset: Logarithmic vertical scale of (a) for clarity. (b)Corresponding cumulative volume fraction for the activities.*

Supplementary methods 2 Sound Levels and CPET Mask Attenuation

To determine the extent to which the CPET masks attenuated the sound level of participant voices, a stable sound source (1Khz octave-band noise) was played from a small Bluetooth speaker (34 mm × 22 mm) held in the mouth of a healthy volunteer. A-weighted time averaged (10 seconds) sound levels were recorded in a sound treated room (ambient noise levels approximately 20 dBA (LeqA10s)) at 30cm distance using a Class 1 sound level metre (Bruel & Kjaer Model 2270) calibrated with a 1kHz 94 dBA calibration tone (using a Bruel & Kjaer Model 4230 calibrator). The intensity of the sound source was modulated upwards in roughly even increments from approximately 45 dBA (LeqA10s) to approximately 80 dBA (LeqA10s), which was as loud as the Bluetooth speaker could produce. At each level of intensity, the sound level was recorded with and without CPET masks of various sizes *in situ*. The resulting levels of attenuation at the different levels of intensity are shown for the four mask sizes used in the study (see Supplementary Figure 5). These data were used to revise the target loudness levels required when the masks were being worn by participants.

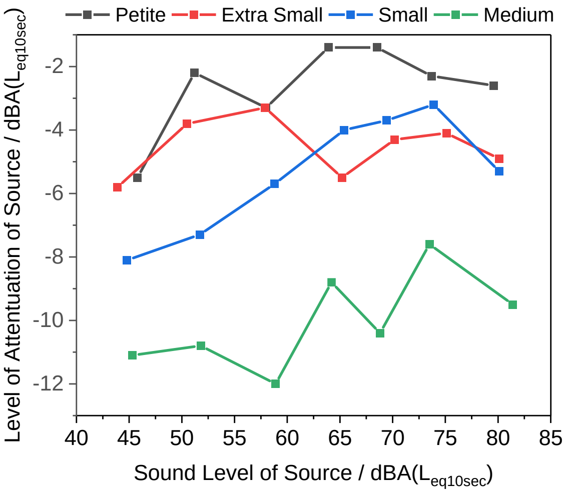

Supplementary Figure 5 ***CPET mask sound attenuation.*** *Sound pressure attenuation in dBA loss for CPET masks sized medium to extra small.*

During the speaking component of the two-stepped exercise test, sound levels were recorded using a Class 2 sound level meter (RS Pro, Model RS-8852) at a distance of 30 cm from the participant's mouth. A-weighted time averaged (30 seconds) sound levels recorded during both aerosol measurements when the participant was speaking are shown in Supplementary Figure 6. Across all participants (n=2 participants data unavailable due to sampling error), a median sound level of 70.0 (IQR 69.0 – 71.3) dBA was determined. Accounting for the size-dependent attenuation of between -2 and -8 dBA (for a source sound level of approximately 75 dBA) shown above for CPET masks, we expect that data recorded in this study was within the desired sound level range of 70-80 dBA.

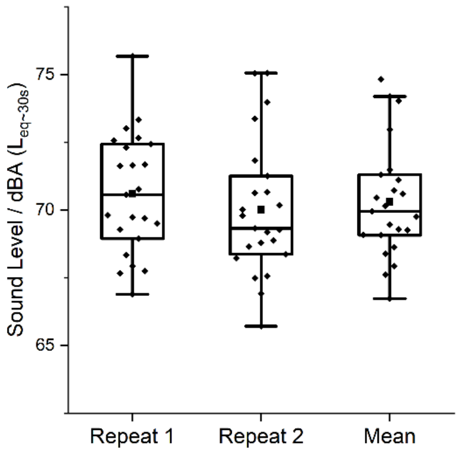

Supplementary Figure 6. ***Sound levels during speaking measurements.*** *Box-plot of time-averaged A-weighted sound levels recorded whilst speaking during each aerosol measurement (Repeat 1 and Repeat 2) and the mean of both values across n=25 participants. Boxes indicate mean, median and IQR, whiskers indicate range (data within 1.5 IQR).*

Supplementary methods 3 Size Distribution, Particle Number, Particle Mass and Mass Emission Data

	Particle Number Concentration / cm^{-3}				Particle Mass Concentration / $\mu g\ m^{-3}$			
	Rest	Speaking 70-80 dBA	Vigorous Exercise	Very Vigorous Exercise	Rest	Speaking 70-80 dBA	Vigorous Exercise	Very Vigorous Exercise
Mean	0.03	0.25	0.12	0.28	0.02	0.52	0.13	0.38
Median	0.03	0.26	0.12	0.24	0.02	0.40	0.17	0.42
25%	0.01	0.21	0.06	0.16	0.01	0.25	0.07	0.24
75%	0.07	0.31	0.25	0.50	0.05	1.22	0.34	0.66
Bottom Whisker	0.002	0.14	0.01	0.06	0.001	0.10	0.02	0.07
Top Whisker	0.16	0.50	0.87	1.80	0.29	9.08	1.08	1.84
n	25	25	25	25	25	25	25	25

Supplementary Table 1. Summarised particle number and mass concentrations for resting, speaking at 70-80dBA, and vigorous exercise and very vigorous exercise across n=25 participants, recorded via APS-mask

Activity	Mode 1			Mode 2			r^2
	N / cm^{-3}	D_p / µm	σ	N / cm^{-3}	D_p / µm	σ	
Rest	0.008 ± 0.002	0.57 ± 0.04	1.32 ± 0.02	-	-	-	0.84
Speaking 70-80 dBA	0.196 ± 0.02	0.69 ± 0.01	1.38 ± 0.01	0.037 ± 0.01	1.74 ± 0.10	1.27 ± 0.02	0.96
Vigorous Exercise	0.115 ± 0.02	0.59 ± 0.06	1.56 ± 0.03	-	-	-	0.86
Very Vigorous Exercise	0.261 ± 0.02	0.71 ± 0.02	1.50 ± 0.01	-	-	-	0.94

Supplementary Table 2. Log-Normal fitting parameters and associated standard errors (SE) for size distributions obtained from breathing at rest, speaking at 70-80 dBA, vigorous exercise and very vigorous exercise, across n=25 participants.

	Particle Number Emission Rate / s^{-1}				Particle Mass Emission Rate / ng s^{-1}			
	Rest	Speaking 70-80 dBA	Vigorous Exercise	Very Vigorous Exercise	Rest	Speaking 70-80 dBA	Vigorous Exercise	Very Vigorous Exercise
Mean	5.3	59.0	120.9	490.8	0.0036	0.1240	0.1341	0.6664
Median	6.5	58.5	145.0	624.9	0.0030	0.0922	0.2068	0.6815
25%	2.4	43.1	45.8	229.6	0.0014	0.0611	0.0528	0.3117
75%	13.8	97.5	285.1	1003.2	0.0134	0.2339	0.3648	1.2842
Bottom Whisker	0.5	19.1	8.9	84.4	0.0001	0.0301	0.0039	0.0825
Top Whisker	28.0	306.2	1153.2	3379.4	0.0383	1.3108	1.1507	4.2091
n	25	25	25	25	25	25	25	25

Supplementary Table 3. Summarised total particle number and mass emission rates for resting, speaking at 70-80 dBA, and vigorous exercise and very vigorous exercise across n=25 participants, recorded via APS-mask.

Supplementary methods 4 Minute Ventilation Compared With and Without APS Sampling

Minute ventilation (L min^{-1}) for all participants was compared with and without concomitant aerosol sampling via APS-mask (Supplementary Figure 7). During periods without APS-mask sampling, the bung was placed in the sampling hole of the modified CPET facemask. Paired t-tests were used to assess any differences in minute ventilation resulting from the presence or absence of APS sampling. At rest, VE with APS-mask sampling was lower than without APS-mask sampling (11.4 ± 3.9 vs 14.1 ± 3.3 L min^{-1}, respectively, $t(24)=4.1$ $p<0.001$). During vigorous exercise, VE with APS-mask sampling was no different than without APS-mask sampling (62.6 ± 17.9 vs 61.4 ± 15.9 L min^{-1}, respectively, $t(24)=1.9$ $p=0.071$). During very vigorous exercise, VE with APS-mask sampling was higher than without APS-mask sampling (113.6 ± 38.7 vs 95.2 ± 30.2 L min^{-1}, respectively, $t(24)=6.2$ $p<0.001$). The very vigorous exercise finding is likely to relate to the APS-mask sampling occurring after the period without APS-mask sampling, in the context of activity above the anaerobic threshold, where minute ventilation rises dramatically.

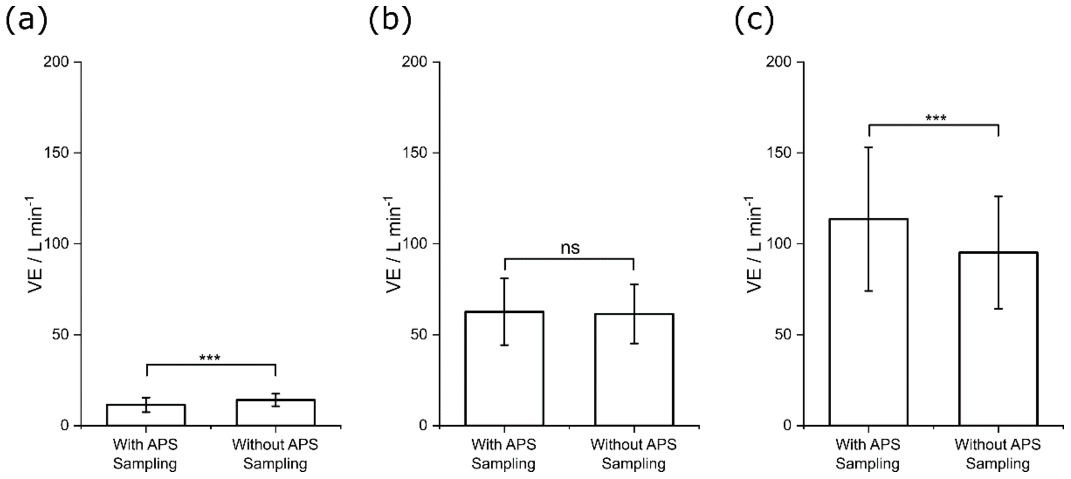

Supplementary Figure 7 ***Effect of aerosol sampling on ventilation.*** *Mean minute Ventilation (VE) for n=25 participants (error bars indicate standard deviation) at rest (a), during vigorous exercise (b) and very vigorous exercise (c), compared with and without APS-mask sampling. Error bars indicate SD, *** indicates p<0.001.*

Supplementary methods 5 Comparisons of Respiratory Particle Emissions by Sex

Using independent samples t-test analyses for sex (males vs females, see Supplementary Figure 8), there were no consistent differences in aerosol particle production either in aerosol particle number concentration, mass concentration or aerosol emission rates and consistent with our

recent study on respiratory aerosol production from singers[1]. Particle mass concentrations were greater in females during speaking (p=0.03) but significance was not demonstrated when testing was controlled for multiple comparisons.

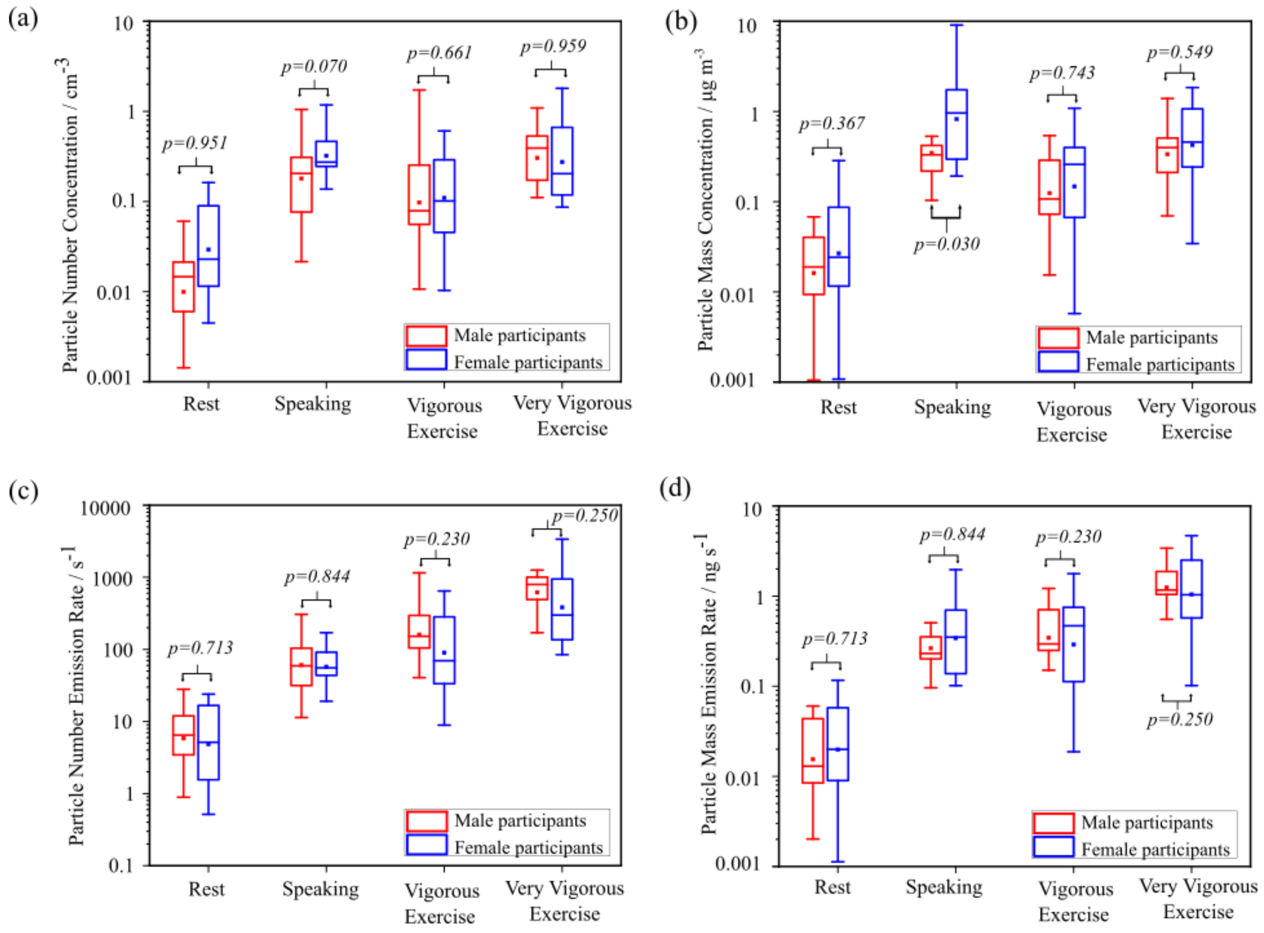

Supplementary Figure 8 ***Comparing aerosol production during breathing at rest, speaking, vigorous exercise, and very vigorous exercise across sex.*** *n=25 participants. Box and whisker plots showing (a) Number concentration, (b) Mass concentration, (c) Particle number emission rate and (d) Particle mass emission rate, for 13 males (blue), 12 females (red), respectively, across activities.*

References

1 Gregson, F. K. A. *et al.* Comparing aerosol concentrations and particle size distributions generated by singing, speaking and breathing. *Aerosol Science and Technology* **55**, 681-691, doi:10.1080/02786826.2021.1883544 (2021).

2 Gregson, F. K. *et al.* Analytical challenges when sampling and characterising exhaled aerosol. *Aerosol Science and Technology*, 1-16 (2021).

3 Johnson, G. *et al.* Modality of human expired aerosol size distributions. *Journal of Aerosol Science* **42**, 839-851 (2011).
