# Supplementary material for: A comparison of respiratory particle emission rates at rest and while speaking or exercising
Source: Commun Med (Lond). 2022 Apr 19;2:44. doi: 10.1038/s43856-022-00103-w (PMC9053213; doi:10.1038/s43856-022-00103-w)
Supplement: Supplementary file 3 — Reporting Summary [file 43856_2022_103_MOESM3_ESM.pdf]

nature research

Corresponding author(s): Dr Christopher M Orton, Professor Jonathan P. Reid.

Last updated by author(s): Feb 18, 2022

Reporting Summary

Nature Research wishes to improve the reproducibility of the work that we publish. This form provides structure for consistency and transparency in reporting. For further information on Nature Research policies, see our Editorial Policies and the Editorial Policy Checklist.

Statistics

For all statistical analyses, confirm that the following items are present in the figure legend, table legend, main text, or Methods section.

n/a	Confirmed	
☐	☒	The exact sample size (n) for each experimental group/condition, given as a discrete number and unit of measurement
☐	☒	A statement on whether measurements were taken from distinct samples or whether the same sample was measured repeatedly
☐	☒	The statistical test(s) used AND whether they are one- or two-sided<br>*Only common tests should be described solely by name; describe more complex techniques in the Methods section.*
☐	☒	A description of all covariates tested
☐	☒	A description of any assumptions or corrections, such as tests of normality and adjustment for multiple comparisons
☐	☒	A full description of the statistical parameters including central tendency (e.g. means) or other basic estimates (e.g. regression coefficient) AND variation (e.g. standard deviation) or associated estimates of uncertainty (e.g. confidence intervals)
☐	☒	For null hypothesis testing, the test statistic (e.g. F, t, r) with confidence intervals, effect sizes, degrees of freedom and P value noted<br>*Give P values as exact values whenever suitable.*
☒	☐	For Bayesian analysis, information on the choice of priors and Markov chain Monte Carlo settings
☒	☐	For hierarchical and complex designs, identification of the appropriate level for tests and full reporting of outcomes
☒	☐	Estimates of effect sizes (e.g. Cohen's d, Pearson's r), indicating how they were calculated

Our web collection on statistics for biologists contains articles on many of the points above.

Software and code

Policy information about availability of computer code

Data collection	No software was utilised for data collection
Data analysis	MLwiN Version 3.05 was used for the multilevel analysis. (Charlton, C., Rasbash, J., Browne, W.J., Healy, M. and Cameron, B. (2020) MLwiN Version 3.05. Centre for Multilevel Modelling, University of Bristol.). MLwiN has a point and click interface so there is no specific code to make available and the software is freely available to UK academics.

For manuscripts utilizing custom algorithms or software that are central to the research but not yet described in published literature, software must be made available to editors and reviewers. We strongly encourage code deposition in a community repository (e.g. GitHub). See the Nature Research guidelines for submitting code & software for further information.

Data

Policy information about availability of data

All manuscripts must include a data availability statement. This statement should provide the following information, where applicable:

- Accession codes, unique identifiers, or web links for publicly available datasets
- A list of figures that have associated raw data
- A description of any restrictions on data availability

Source data underlying the figures and the raw data used in the analysis have been made publicly available in the BioStudies database, https://www.ebi.ac.uk/biostudies/, under accession ID S-BSST691.

Field-specific reporting

Please select the one below that is the best fit for your research. If you are not sure, read the appropriate sections before making your selection.

☒ Life sciences ☐ Behavioural & social sciences ☐ Ecological, evolutionary & environmental sciences

For a reference copy of the document with all sections, see nature.com/documents/nr-reporting-summary-flat.pdf

Life sciences study design

All studies must disclose on these points even when the disclosure is negative.

Sample size	Sample size calculations were built into the study protocol based on the data observed in a previous study (Perform 1) for similar measurements and designed to find differences of magnitude 2.5 (on the raw scale) between groups based on a power of 0.9 and building in potential dropouts
Data exclusions	No data exclusions were required in the analysis. 6 participants were unable to complete a second trial of the very vigorous exercise however the statistical analysis was based on averages over activity for participants so for these participants the average was therefore based on 1 rather than 2 data points.
Replication	Measurements of particle counts were made in duplicate, as outline in the methods.
Randomization	The study was non-randomised
Blinding	The study was not blinded

Reporting for specific materials, systems and methods

We require information from authors about some types of materials, experimental systems and methods used in many studies. Here, indicate whether each material, system or method listed is relevant to your study. If you are not sure if a list item applies to your research, read the appropriate section before selecting a response.

Materials & experimental systems

n/a	Involved in the study	
☒	☐	Antibodies
☒	☐	Eukaryotic cell lines
☒	☐	Palaeontology and archaeology
☒	☐	Animals and other organisms
☐	☒	Human research participants
☒	☐	Clinical data
☒	☐	Dual use research of concern

Methods

n/a	Involved in the study	
☒	☐	ChIP-seq
☒	☐	Flow cytometry
☒	☐	MRI-based neuroimaging

Human research participants

Policy information about studies involving human research participants

Population characteristics	Twenty-five participants (13 males, 12 females), with a mean age of 36.4 years, (SD ± 14.9 years, range 19-72) and normal body mass index (BMI) at 23.8 kg m-2 (SD ± 4.1). Participants exhibited a broad range of athletic capability, mean peak oxygen uptake per kg (VO2 kg-1) 42.4 ml kg-1 min-1 (SD ± 11.01, range 26 to 65).
Recruitment	Participants were recruited via professional and personal performance networks.
Ethics oversight	Public Health England Research Ethics and Governance of Public Health Practice Group (PHE REGG, NR0221).

Note that full information on the approval of the study protocol must also be provided in the manuscript.
